# Supplementary material for: A non-equilibrium formulation of food security resilience
Source: R Soc Open Sci. 2017 Jan 18;4(1):160874. doi: 10.1098/rsos.160874 (PMC5319352; doi:10.1098/rsos.160874)
Supplement: Appendices [file rsos160874supp1.pdf]

Non-equilibrium resilience – Supporting Information

Appendix A: Per capita kilocalorie database

See file kcal.csv for per capita kilocalorie availability database. Values for Belgium 1961-1999 and Luxembourg 1961-1999 are taken from combined Belgium-Luxembourg data. Values for Czech Republic and Slovakia 1961-1990 are taken from combined Czechoslovakia data. Values for Serbia and Montenegro 1991-2005 are taken from combined Serbia-Montenegro data. Values for Bosnia-Herzegovina, Croatia, Macedonia, Montenegro, Serbia, and Slovenia 1961-1990 are taken from Yugoslav SFR data. Values for Belarus, Estonia, Latvia, Lithuania, Russia, and the Ukraine 1961-1990 are taken from USSR data.

Appendix B: Statistical properties database

See file results.csv for statistical properties of country time series, including candidate ARMA models and the corresponding AIC scores.

Appendix C: Countries ranking

	**Decreasing Level**	**Decreasing Trend**	**Increasing Rel. Volatility**	**Increasing Persistence**
1	Turkey	China	Egypt	Sweden*
2	Montenegro	Algeria	China	Benin***
3	Israel	Egypt	Algeria	Belgium***
4	Bosnia-Herzegovina	Saudi Arabia	Brazil	Luxembourg***
5	Belarus	Burkina Faso	Costa Rica	Chile***
6	Serbia	Iran	El Salvador	Thailand**
7	Macedonia	Morocco	South Korea	Fiji*
8	Ukraine	Mali	Iran	Ecuador***
9	Croatia	South Korea	Morocco	New Zealand*
10	Russia	Cuba	Honduras	United Kingdom*
⋮	⋮	⋮	⋮	⋮
152	Cambodia	Central African Republic	Swaziland	South Korea***
153	Burkina Faso	Namibia	Slovenia	Macedonia
154	East Timor	Macedonia	Macedonia	Estonia**
155	Chad	Chad	Argentina	Serbia*
156	Haiti	Zambia	Ukraine	Cambodia
157	Mozambique	Bulgaria	Uganda	Kuwait***
158	Angola	Serbia	Bosnia-Herzegovina	Iraq**
159	Djibouti	Madagascar	Finland	Sierra Leone**
160	Myanmar	Slovakia	Czech Republic	Saudi Arabia***
161	Ethiopia	Afghanistan	Croatia	Angola***

TABLE I. Best and worst performers by statistical feature, per capita daily kcal availability, 1961-2011. Stars in the last column indicate the p-value for autocorrelation of Δ_t according to the Ljung-Box test (***Significant at $p < 0.01$; **significant at $p < 0.05$; *significant at $p < 0.1$).

Appendix D: Cross-country regressions

We fit the simple linear model

$$\rho, \pi = a + b * (trade) + \sum_{k=1}^{m} c_i X_i + \epsilon_i \tag{D1}$$

to test the hypothesis that trade openness ($trade$) is positively correlated to volatility relative to trend (ρ) and negatively correlated to persistence (π), given a vector of control variables $X_1....X_m$ representing economic resources, human capital stocks, and political participation (see Table II). We also include the same determinants in models predicting mean kcal level and growth rate g. In all models, errors are independent and identically distributed with mean zero and standard deviation σ; error variances are heteroskedastic, and Huber-White standard errors are used in estimation. We also specify the same models using the subset of developing countries (as classified by the United Nations in 2012) only (Table III); results are similar.

	$kcal$ (level)	g (trend)	ρ (volatility relative to trend)	π (persistence)
trade	-0.382 (0.908)	-0.046** (0.018)	-0.083 (0.068)	-0.001 (0.001)
gdppc	0.010*** (0.002)	-0.001 (0.001)	0.001 (0.001)	-0.001* (0.001)
literacy	1006.058*** (185.671)	4.862 (6.395)	-64.301 (59.835)	0.145 (0.112)
polity	16.185** (6.206)	-0.509*** (0.161)	-0.248 (0.617)	-0.009* (0.004)
constant	1596.485 (128.69)	9.360 (5.204)	37.440 (36.119)	-0.124 (0.088)
r^2	0.653	0.107	0.010	0.084

TABLE II. Results of all country models predicting level, trend, volatility relative to trend, and persistence of per capita daily kcal availability. Standard errors in parentheses. ***Significant at p¡0.01; **significant at p¡0.05; *significant at p¡0.1. Sources: trade openness (sum of exports and imports of goods and services measured as a share of gross domestic product) [?]; GDP per capita, literacy [?]; polity10 (degree of democracy and autocracy) [?].

	kcal (level)	g (trend)	ρ (relative volatility)	π (persistence)
trade	-0.072 (1.151)	-0.039** (.022)	0.039 (0.089)	-0.001 (0.001)
gdppc	0.017*** (0.003)	-0.001 (.001)	-0.001 (0.001)	-0.001 (0.001)
literacy	700.963*** (177.437)	8.193 (6.852)	-62.530 (59.597)	0.071 (0.119)
polity	15.620** (7.379)	-0.500*** (0.183)	-0.881(1.093)	-0.010** (0.004)
constant	1785.826 (124.801)	7.158 (5.442)	30.299 (33.334)	-0.105 (0.089)
r^2	0.512	0.085	0.018	0.064

TABLE III. Results of developing country only models predicting level, trend, relative volatility, and persistence of per capita daily kcal availability. Standard errors in parentheses. ***Significant at $p < 0.01$; **significant at $p < 0.05$; *significant at $p < 0.1$. Same sources as Table II.

Appendix E: Illustration of resilience and resistance with synthetic data

Below we illustrate our concepts of resilience and resistance using synthetic data (300 realizations) generated with different ARIMA(1,1,0) model with identical trends but varying autoregressive coefficient β_1 (Fig. 1) or absolute volatility σ (Fig. 2).

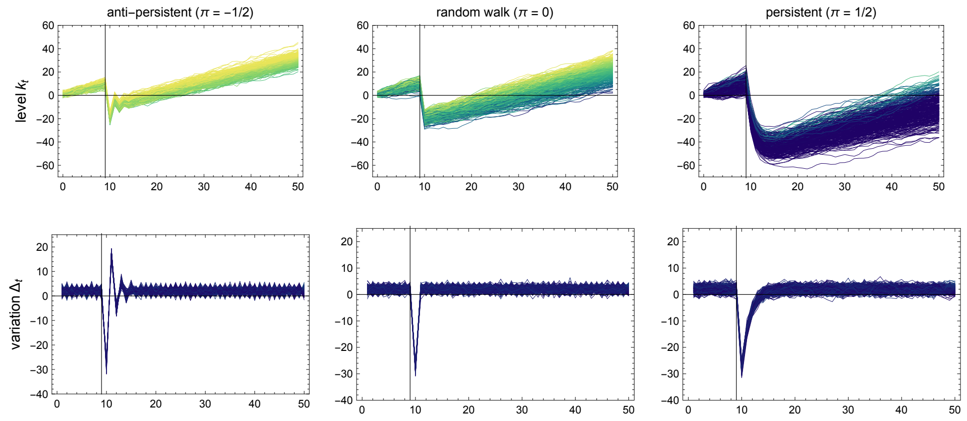

FIG. 1. Resilient and non-resilient trajectories, constructed with synthetic data. The actor in the left column, with $\pi < 0$ and a non-deteriorating long-term trend, is resilient. The actor in the right column, with $\pi > 0$, exhibits persistent, and thus non-resilient, behavior. The actor in the middle column corresponds to the marginal case of random walk with drift.

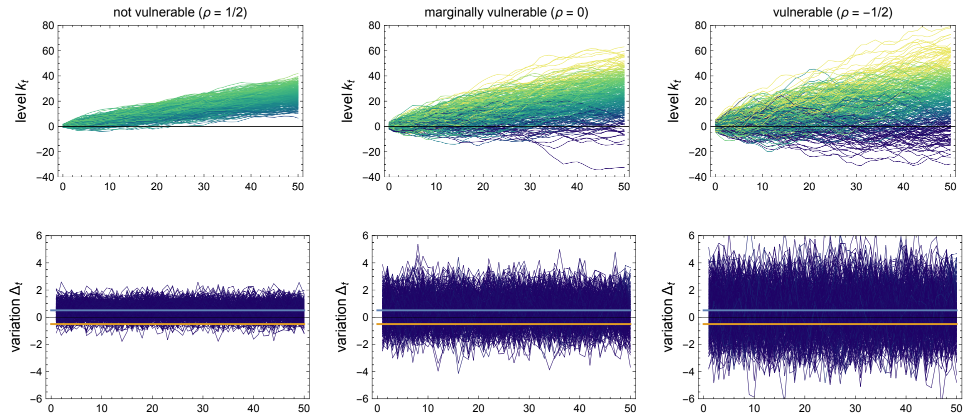

FIG. 2. Resistant vs. volatile trajectories. The actor in the left column, with $\rho \geq 0$ and a non-deteriorating long-term trend, is resistant to shocks. The second and third rows illustrate that volatile actors experience much stronger fluctuations. Here the blue line indicates the mean trend g, while the orange line gives the opposite trend $-g$.
